# The Value of Neutrophil Gelatinase-Associated Lipocalin Receptor as a Novel Partner of CD38 in Chronic Lymphocytic Leukemia: From an Adverse Prognostic Factor to a Potential Pharmacological Target?

**DOI:** 10.3390/biomedicines11092335

**Published:** 2023-08-22

**Authors:** Brigitte Bauvois, Elise Chapiro, Claire Quiney, Karim Maloum, Santos A. Susin, Florence Nguyen-Khac

**Affiliations:** 1 Centre de Recherche des Cordeliers, Sorbonne Université, Université Paris Cité, Inserm UMRS1138, Drug Resistance in Hematological Malignancies Team, F-75006 Paris, France; elise.chapiro@aphp.fr (E.C.); karim.maloum@aphp.fr (K.M.); santos.susin@sorbonne-universite.fr (S.A.S.); florence.nguyen-khac@aphp.fr (F.N.-K.); 2 Groupe Hospitalier Pitié-Salpêtrière, Assistance Publique-Hôpitaux de Paris, Service d’Hématologie Biologique, F-75013 Paris, France; c.quiney@filo-leucemie.org

**Keywords:** CLL, CD38, NGAL-R, SLC22A17, relapse, remission, survival, bispecific antibody

## Abstract

Chronic lymphocytic leukemia (CLL) is characterized by the accumulation of neoplastic B lymphocytes that escape death, and correlates with the expression of negative prognostic markers such as the CD38 antigen. Although certain new drugs approved by the US Food and Drug Administration improve the clinical outcome of CLL patients, drug resistance and disease relapse still occur. Like CD38, neutrophil gelatinase-associated lipocalin receptor (NGAL-R) is frequently overexpressed in CLL cells. Here, we evaluated the concomitant surface expression of NGAL-R and CD38 in leukemic blood cells from 52 CLL patients (37 untreated, 8 in clinical remission, and 7 relapsed). We provide evidence of a positive correlation between NGAL-R and CD38 levels both in the interpatient cohorts (*p* < 0.0001) and in individual patients, indicating a constitutive association of NGAL-R and CD38 at the cell level. Patients with progressing CLL showed a time-dependent increase in NGAL-R/CD38 levels. In treated CLL patients who achieved clinical remission, NGAL-R/CD38 levels were decreased, and were significantly lower than in the untreated and relapsed groups (*p* < 0.02). As NGAL-R and CD38 participate in CLL cell survival, envisioning their simultaneous inhibition with bispecific NGAL-R/CD38 antibodies capable of inducing leukemic cell death might provide therapeutic benefit for CLL patients.

## 1. Introduction

Chronic lymphocytic leukemia (CLL) is defined as a slow-developing disease caused by a peripheral accumulation of CD5^+^ B lymphocytes in the peripheral blood, bone marrow, and secondary lymphoid organs [1]. The leukemic cells (which are mostly quiescent) mainly accumulate because they are unable to develop a cell death program—even though proliferating pools are found in the bone marrow and lymph nodes [1]. Besides the conventional chemotherapeutic agents, the main drugs currently prescribed are signaling inhibitors targeting B cell receptor (BCR)-associated kinase (i.e., Bruton’ s tyrosine kinase (BTK) inhibitors ibrutinib and acalabrutinib), or the antagonist of the B-cell lymphoma-2 (Bcl-2) anti-apoptotic protein (venetoclax) [2]. In a few cases, the combination of classical agents with CD20 monoclonal antibodies (mAbs) (i.e., rituximab and obituzumab) is also proposed [2]. However, these therapies are often accompanied by adverse effects or favored mutations associated with drug resistance, as well as non-genomic resistance [3,4,5,6,7,8]. Thus, treatment of CLL remains a challenge in the clinic, and novel therapies are needed. In this way, the identification of new molecules that could predict the prognosis and achieve treatment in CLL is of general interest.

CLL cells are characterized by high expression of CD5, CD19 and CD23 antigens [9]. Other surface markers may have prognostic importance in CLL, such as CD38, which plays a dual role as a receptor and ectoenzyme (bearing both cyclase and hydrolase activities) [9]. CD38 interacts with the CD31 antigen constitutively expressed by endothelial cells, allowing leukocyte adhesion and migration [9]. High CD38 expression in CLL is associated with a hyperproliferative phenotype, and is an independent adverse prognostic factor [9,10]. CD38-mediated CLL cell survival occurs through the activation of an AKT/SYK/Mcl-1 signaling [11]. Preclinical studies (ex vivo experiments and CLL xenograft models) have demonstrated the antitumor efficacy of the CD38 mAb daratumumab in monotherapy or combination therapy with ibrutinib [12]. Further, CD38 mAbs have recently emerged as promising treatment options in CLL [2,13]. The combination of daratumumab with ibrutinib in CLL patients—untreated, refractory, or relapsed with a poor prognosis (e.g., 17p deletion and/or *TP53* mutation)—is being studied in three active phase Ib/II clinical trials (NCT03447808, NCT03734198, NCT04230304). The initial results of the NCT03734198 study showed no significant objective responses compared with ibrutinib alone [14]. A recent strategy of immunotherapy uses bispecific Abs (Bi-Abs), which have more advantages than monospecific Abs. For instance, a CD38/CD3 Bi-Ab (ISB-1342) that targets tumor cells (via CD38) and immune effector T cells (via CD3) is in phase II for relapsed/refractory multiple myeloma (R/R MM) (NCT033309111); the putative efficacy of ISB-1342 in CLL remains to be tested. Given the widespread expression of CD38 in immune populations (e.g., in plasma cells, T and NK cells, myeloid precursor cells, and dendritic cells) [15], CD38/CD3 Bi-Abs may substantially increase the risk of worse syndromic events. To further enhance therapeutic effects, an alternative approach aims at optimizing Bi-Abs which recognize two tumor antigens on tumor cells co-expressing both antigens [16]. Thus, a CD19/CD47 Bi-Ab (TG-1801) has recently entered into clinical development for CLL treatment (NCT04806035).

Neutrophil gelatinase-associated lipocalin (NGAL) is a secreted protein which is capable of binding low-molecular-weight ligands and of capturing siderophores that bind iron with high affinity [17]. NGAL interacts as ligand with a cell surface receptor NGAL-R (also known as SLC22A17) that is able to bind NGAL alone or is bound to a siderophore and iron [17]. NGAL-R belongs to the solute carrier 22 (SLC22) family of organic ion transporters [17]. Recent work from our group showed that (i) serum levels of NGAL (derived from circulating CLL cells) are significantly elevated in untreated CLL patients [18], and (ii) NGAL-R (absent in normal B, T and myeloid compartments) is frequently overexpressed by CLL cells from untreated patients [18]. Moreover, we presented first evidence that the NGAL-R/NGAL complex enhances the survival of CLL cells by activating an SRC/STAT3/Mcl-1 signaling pathway [18].

So far, there are no data on the association of CD38 and NGAL-R during the progression of CLL and following therapeutic treatment. The goal of the present study was to determine whether blood CLL cells co-express CD38 and NGAL-R, and to this end we assessed the levels of CD38 and NGAL-R in CLL cells at the same time point for each patient, as a function of disease severity and treatment.

## 2. Materials and Methods

### 2.1. Patients

Peripheral blood was collected from 52 patients (during the period from 2016 to 2018) diagnosed with CLL. Patients were diagnosed according to the International Workshop on CLL (IWCLL) criteria, and staged according to the Binet system [19]. The main exclusion criteria included neoplastic disease other than CLL, diabetes, hepatic fibrosis, and renal or lung disease. Patients were selected based on the following criteria: lymphocytosis, lymphocyte morphology, clinical features, CD5^+^/CD19^+^ immunophenotype, and *IGHV* mutation status. Progressive CLL was characterized by expansion of the neoplastic clone and extravascular accumulation in lymphoid tissues, the bone marrow, and other organs, giving rise to immune dysfunction, lymphadenopathy, splenomegaly and hematopoietic failure. Clinical remission is when the bone marrow contains fewer than 5% blast cells, the blood cell counts return to within normal limits, and there are no signs or symptoms of the disease. Deletions of 17p13, 11q22, 13q14 and trisomy 12 were detected using fluorescence in situ hybridization (FISH) with the Metasystems XL DLEU/LAMP/12cen and XL ATM/TP53 Multi-Color Probe Kits (MetaSystems, Compiègne, France). The patients’ clinical characteristics are summarized in Table 1.

### 2.2. CLL Cell Separation and Flow Cytometry

Peripheral blood mononuclear cells were isolated from blood using Ficoll-Hypaque density gradient (1.077 g/mL) centrifugation. Freshly isolated cells were stained and analyzed immediately in the flow cytometry assay, using anti-CD19 (clone 4G7, mIgG1; Santa-Cruz, Heidelberg, Germany), anti-CD5 (clone 205919, mIgG1; R&D Systems, Abingdon, UK), anti-CD38 (clone HB7, mouse IgG1; BD Biosciences, LePont de Claix, France), anti-NGAL-R (clone 16315, rabbit IgG; CliniSciences, Nanterre, France), and appropriate isotype controls (Santa-Cruz). Stained cells were analyzed with a Coulter Epics XL flow (Beckman-Coulter, Les Ullis, France) or a FACSCanto II flow (BD Biosciences, Le Pont de Claix, France) cytometer. Data were analyzed using LYSYS (Beckman-Coulter) or FloJo (version 7.0, BD Biosciences, Le Pont de Claix, France) software.

### 2.3. Statistics

Mann–Whitney tests, Student’s *t*-tests and one-way ANOVA were performed using GraphPad Prism software (version 7.0, GraphPad Software, La Jolla, CA, USA). For greater stringency, all tests were two-tailed. Significance levels were defined as * *p* < 0.05, ** *p* < 0.01, and **** *p* < 0.0001.

### 2.4. Ethics Statement

In line with the ethical tenets of the Declaration of Helsinki, the study protocol was approved by the ethical board on human experimentation at the Pitié-Salpêtrière Hospital on 21 May 2014 (CPPIDF6, Paris, France). The patients provided their written and informed consent to participation in the study.

## 3. Results

### 3.1. Prognostic Relevance of NGAL-R and CD38 Co-Expression

**Figure 1:**
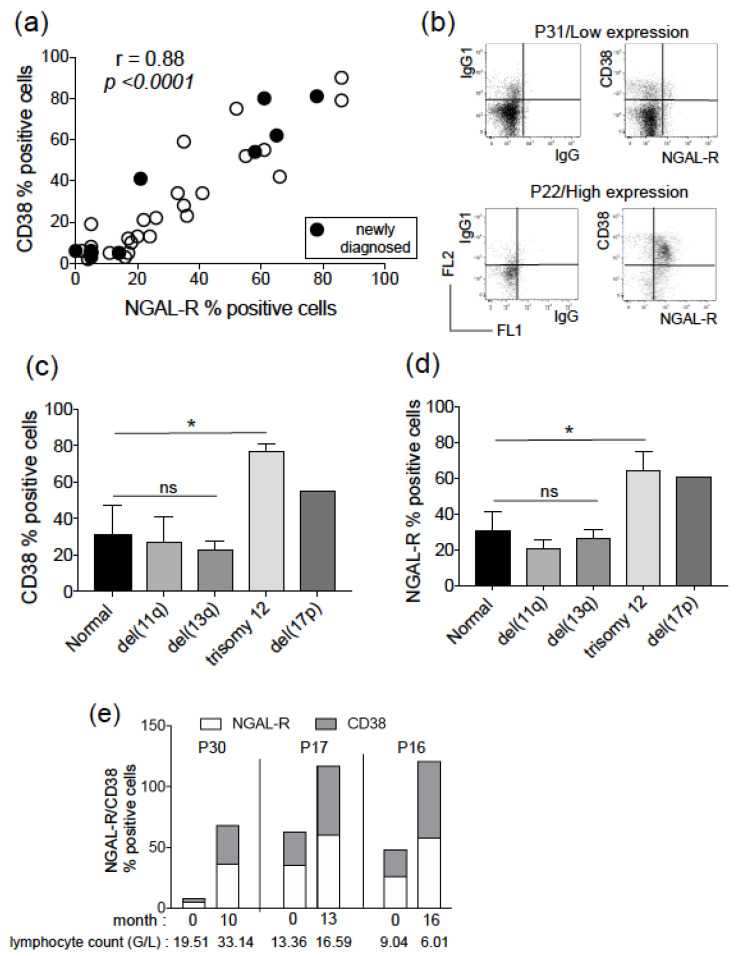
Correlation of surface expression of CD38 and NGAL-R in CLL cells from untreated patients. (**a**) The plot compares the percentage surface CD38 and NGAL-R co-expression on CLL cells derived from 37 untreated CLL patients (10 newly diagnosed and 27 in follow-up). Spearman’s correlation coefficient (r) and the *p* value are shown. (**b**) Representative cytograms of CLL cells from 2 untreated patients (P31, P22), stained with rabbit IgG-FITC/mIgG1-PE, and NGAL-R-FITC/CD38-PE Abs. (**c**) CD38 and (**d**) NGAL-R surface levels were determined in CLL cells from patients without (normal, 4 patients) or with del(11q) (4 patients), del(13q) (27 patients), trisomy 12 (4 patients) and del(17p) (1 patient). The data are presented as mean ± SEM; ANOVA values are shown. * *p* < 0.05; not significant (ns). (**e**) Levels of NGAL-R and CD38, and lymphocyte count (G/L) in 3 untreated, stage A CLL patients (P16, P17, P30) over months after first analysis. Patient P16 progressed to stage B at month 16.

We measured surface levels of NGAL-R and CD38 (at the same time point for each patient) in CD19^+^/CD5^+^ blood samples from 37 untreated CLL patients (including 10 newly diagnosed patients). A significant correlation was observed between the percentage positivity of CD38 and NGAL-R within the total cohort (r = 0.88, *p* < 0.0001) (Figure 1a). Analysis of NGAL-R/CD38 co-expression showed no significant differences between the group of newly diagnosed patients and the group of CLL follow-up patients (Figure 1a). In addition to the interpatient relationship, a positive correlation between CD38 and NGAL-R was observed on CLL cells within individual patients, as illustrated in Figure 1b by two representative experiments: patient P31 exhibited low NGAL-R/CD38 co-expression (<10%), while patient P22 showed high NGAL-R/CD38 co-expression (>80%), which strongly suggests the colocalization of the two proteins at the cell level. When a 30% cut-off for NGAL-R/CD38 co-expression was used, no significant difference was observed between (NGAL-R/CD38)^low^ and (NGAL-R/CD38)^high^ groups regarding sex, age, lymphocyte count, Binet stage, and IGHV mutational status. Enhanced CD38 expression is associated with CLL cells bearing trisomy 12 or del(17p) [20,21,22]. Our analysis confirmed and completed these data with significantly elevated NGAL-R/CD38 levels found in trisomy 12 cells compared to CLL cells without or with del(11q) and del(13q) (Figure 1c,d). A similar increase in NGAL-R/CD38 levels was also observed in del(17p) CLL cells from patient P5 (Figure 1c,d). The association between NGAL-R/CD38 co-expression and time-to-progression was evaluated in three untreated patients (P16, P17 and P30) with progressive CLL. A longitudinal analysis of CLL cell samples showed that NGAL-R/CD38 levels had increased over time with disease progression, independently of lymphocyte count (Figure 1e).

### 3.2. Correlation of NGAL-R/CD38 Co-Expression with Remission Rate and Outcome

**Figure 2:**
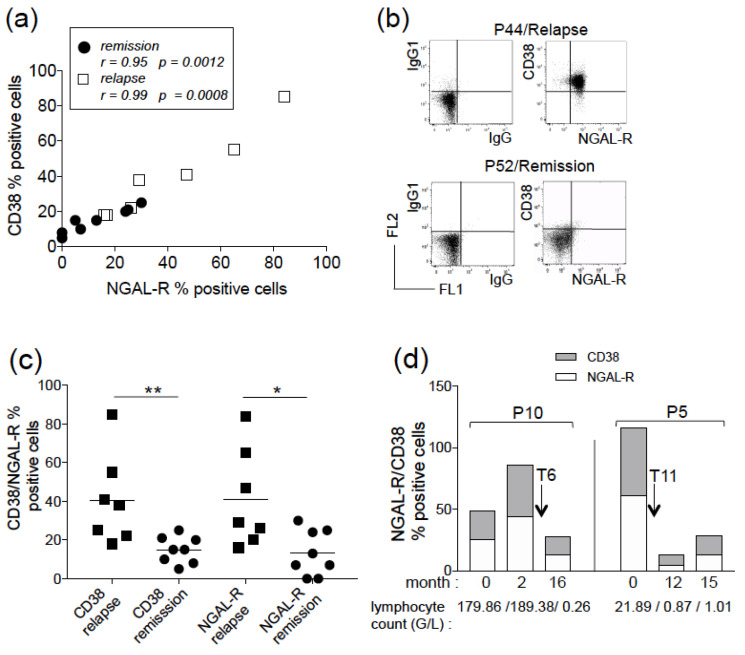
Co-expression profiles of surface NGAL-R and CD38 on CLL cells from patients after therapeutic treatment. (**a**) The plot compares the percentage surface CD38 and NGAL-R co-expression on CLL cells derived from 15 treated CLL patients (8 in remission and 7 relapsed). Spearman’s correlation coefficient (r) and the *p* value are shown. (**b**) Representative cytograms of CLL cells from 2 treated patients (P44/relapse, P52/remission), stained with rabbit IgG-FITC/mIgG1-PE, and NGAL-R-FITC/CD38-PE Abs. (**c**) NGAL-R/CD38 levels were determined in CLL cells from patients at clinical remission (*n* = 8) or relapse (*n* = 7). The data are shown as mean ± SEM, and *p* values are calculated using a Mann–Whitney *U*-test. * *p* < 0.05; ** *p* < 0.01. (**d**) Levels of NGAL-R/CD38 in CLL cells from patient P10 (stage B) at months 0 and 2, treated with FCR at month 6, and at remission (month 16 (patient P49)); levels of NGAL-R/CD38 in CLL cells from patient P5 (stage C) at month 0, treated with RCb at month 11, and at clinical remission (month 12 (patient P45) and month 15). The arrow indicates the beginning of the treatment T. Lymphocyte count (G/L) over months is shown.

As for the untreated patients, we measured a significant positive correlation between the expression of NGAL-R and CD38 in CLL-treated patients, both in the relapsed and clinical remission groups (Figure 2a). Both antigens remained co-expressed at the cell level (Figure 2b); as exemplified in Figure 2b, a high level of NGAL-R^+^/CD38^+^ CLL cells (>80%) was observed for relapsed patient P44, while the proportion of NGAL-R^+^/CD38^+^ CLL cells was low from patient P52 in clinical remission (<10%). The levels of NGAL-R/CD38 were significantly higher in the majority of tumor cell samples from the relapsed group (CD38: mean 40.57% ± 8.85; NGAL-R: mean 41.0% ± 9.63) than in the remission group (CD38: mean 14.88% ± 2.45; NGAL-R: mean 13% ± 4.21) (2.7 and 3.1 times more, respectively; *p* = 0.005 and *p* = 0.019, respectively) (Figure 2c). In contrast, and as expected, the levels of NGAL-R/CD38 in the relapsed group were close to NGAL-R/CD38 levels in the untreated group (CD38: mean 28.86% ± 4.53; mean NGAL-R: 29.97% ± 4.22) (*p* = 0.299 and *p* = 0.303, respectively).

The surface levels of NGAL-R/CD38 were quantified in CLL cells from two patients (P5 and P10) before treatment and at clinical remission (Figure 2d). The level of NGAL-R/CD38 co-expression increased with time in P10′s CLL cells before treatment, and was markedly decreased at remission (month 16 corresponding to 10 months after FCR treatment) (Figure 2d); similarly, the elevated NGAL-R/CD38 levels in untreated patient P5′s CLL cells fell at remission (months 12 and 15, corresponding to 1 and 4 months, respectively, after RCb treatment) (Figure 2d). Thus, the diminution of NGAL-R/CD38 expression appears to be associated with a good treatment response. At the time of our study, we had no patients who had undergone treatment and then relapsed.

NGAL-R/CD38 levels for each treated patient were measured at different time points after the start of the treatment (Table 1). Our data indicate that the remission duration did not seem to influence the rate of NGAL-R/CD38 expression in the interpatient group; for instance, patient P51, in remission for 17 months, exhibited lower NGAL-R/CD38 CLL cell levels (0%/5%) than patient P46, in remission for 3 months (30%/25%). Moreover, in the relapsed group, expression of NGAL-R/CD38 in CLL cells (84%/85% at 24 months after treatment) from patient P44 was markedly higher than in leukemic cells from patients P39 (20%/25% at 48 months after treatment) and P43 (26%/22% at 72 months after treatment). This may be explained by the extreme clinical heterogeneity of CLL. The kinetics of expression of NGAL-R/CD38 on CLL cells during the course of chemotherapy and at relapse would have to be analyzed in individual CLL patients.

## 4. Discussion

The present study establishes the association of NGAL-R with CD38 on the surface of primary CLL cells. The high CD38/NGAL-R co-expression is associated with a poor clinical outcome and represents a potential therapeutic target in CLL.

Reports in the literature highlight common biochemical and functional features for NGAL-R and CD38. First, CD38 and NGAL-R promoters contain CpG islands that can be methylated [23,24]. DNA hypomethylation enhances the expression of CD38 in multiple myeloma [23] and NGAL-R in esophageal squamous cell carcinoma [24]. In CLL, alterations in DNA methylation affect a large number of genes, including CD38 and SLC22 members [25,26], and hypomethylation programming is targeted to regions marked as enhancers and binding sites for various transcription factors, including NF-κB, AP-1 and Runx3 [26]. The CD38 promoter exhibits binding sites for NF-κB and AP-1 which increase the transcription of CD38 via binding to its promoter [27,28]. NGAL-R expression in murine bone marrow cells is upregulated by Runx3 [29]. High Runx3 expression is associated with poor survival in leukemic patients [30]. NGAL-R upregulation depending on Runx3 transcriptional activity in CLL is therefore plausible. Of importance, DNA methylation profiles are modulated during CLL progression, time to first treatment, and response to treatment [31]. These changes in DNA methylation may affect CpG sites of CD38 and NGAL-R genes, including transcription-factor binding sites implicated in the regulation of these proteins. Second, CD38 and NGAL-R mediate—at least in part—convergent prosurvival signalings in CLL. The anti-apoptotic protein Mcl-1 is constitutively expressed in CLL cells, and is involved in the cells’ ability to prevent death [32]. CD38 favors survival in CLL cells by activating an AKT/SYK/Mcl-1 signaling pathway [11], while the binding of NGAL to NGAL-R induces the survival of CLL cells by activating an SRC/STAT3/Mcl-1 signaling [18]. Many signaling pathways related to survival and proliferation are associated with lipid rafts in tumor cells [33]. Since CD38 has a short cytoplasmic tail suggesting it is unable to initiate a signal cascade, its involvement in CD19 and BCR survival signaling has been proposed, as CD38 associates with these two receptors in lipid rafts and enhances BCR signaling in CLL cells [34,35]. Although not yet shown for NGAL-R, several SLC22 receptors associate with lipid rafts [36,37]. As NGAL-R co-exists with CD38 on CLL cells (possibly in lipid rafts), the involvement of NGAL-R in CD38 signaling (and vice versa) might be hypothesized. The neutralizing mAbs daratumumab and GTX85032 targeting CD38 and NGAL-R, respectively, induce ex vivo the death of primary CLL cells [12,18,38]. Therefore, we propose that neutralizing CD38 and NGAL-R Abs may be therapeutically used to interrupt survival-promoting pathways. Moreover, our study endorses a potential therapeutic relevance of CD38/NGAL-R Bi-Abs to target poor-prognosis CLL. Blockade therapies with CD38/NGAL-R Bi-Abs in combination with BTK or Bcl-2 inhibitors already available in clinics might optimize the landscape of CLL therapy.

The consideration of CD38 and NGAL-R as druggable co-targets for CLL immunotherapy is reinforced by the fact that both proteins are involved in tumor metabolism [39,40]. Metabolic changes in tumor cells represent a novel opportunity for therapy approaches [41]. In CLL, CD38, through its enzymatic activity, regulates NAD metabolism which leads to an increase in cytoplasmic Ca^2+^ concentrations, positively influencing proliferation and signaling mediated via chemokine receptors or integrins [42]. Iron, by modulating metabolism, is implicated in the proliferation and survival mechanisms of various cancer cell types, including CLL bearing a *TP53* abnormality [43,44]. By capturing circulating and intracellular free iron [40], NGAL bound to its receptor can regulate iron traffic in tumors [40,45]. For instance, NGAL decreases intracellular iron levels and stimulates the expression of glutathione peroxidase 4 to prevent membrane lipid peroxidation in colon cancer cell lines [46].

To our knowledge, the potential value of CD38/NGAL-R association in the pathogenesis of tumors has not previously been assessed. Our study provides the first evidence that the co-expression of NGAL-R and CD38 is a high-value target in CLL. Beyond CLL, the expression rates of CD38 and NGAL-R are high in other tumors such as acute and chronic myeloid leukemias [47,48,49,50], epithelial carcinomas (esophagus, liver, kidney, endometrial carcinomas) [13,24,51,52,53,54,55] and gliomas [51,56]. Although these studies separately showed elevated expressions of CD38 and NGAL-R, it is very likely that both antigens are co-expressed in these tumors, and therefore suggest that a combination of anti-CD38/NGAL-R Abs may offer new therapeutic options for the management of these cancers. The efficacy of anti-CD38 Abs including daratumumab is already being evaluated in preclinical and clinical trials in these malignancies [13,52,57,58]. Based on this, advances in the area of CD38/NGAL-R Bi-Abs might be of significant consideration for future targeted therapies in these types of cancer, which abnormally express CD38 and NGAL-R.

Finally, the limitations of this study are that a small number of treated patients were studied and relapsed patients were not followed up during the progression of CLL. Moreover, our study was single-centered. Therefore, CD38 and NGAL-R markers should be simultaneously studied on other CLL cohorts in centers which extend diagnostic and therapeutic facilities to CLL patients. However, this pilot study can already form a basis for larger studies on the subject.

## 5. Conclusions

CD38 and NGAL-R represent co-molecular targets with high value in CLL and give good perspectives for the development of innovative therapeutic strategies using Bi-Abs in CLL. CD38/NGAL-R Bi-Abs might have therapeutic potential in CLL patients in relapse or not responding to current therapies.

## Figures and Tables

**Table 1:** Clinical characteristics of chronic lymphocytic leukemia (CLL) patients.

CLL	Sex/ Age	Stage at Diagnosis	Lymphocyte Count G/L at Analysis Time	IGHV	FISH	Karyotype at Diagnosis	Therapy (Analysis Time Point after the Start of Treatment)
1	M/71	A	12.4	M	Tri 12	1 abnormality	None
2	M/63	A	5.51	nd	13q-	Complex	None
3	M/90	A	16.02	M	nd	nd	None
4	M/76	A	48.91	nd	13q-	Normal	None
5	M/87	C	21.89	UM	17p-, 13q-	Normal	None
6	M/66	B	37.22	M	13q-	2 abnormalities	None
7	F/74	A	78	UM	13q-	Normal	None
8	F/78	A	26.84	M	13q-	Normal	None
9	F/68	A	16.15	nd	13q-	1 abnormality	None
10	F/62	B	179.86	UM	13q-	Normal	None
11	F/83	A	64	nd	Normal	Normal	None
12	M/82	A	4.69	nd	11q-, 13q-	Normal	None
13	F/77	A	34.93	UM	13q-	Failure	None
14	M/74	A	30.08	nd	13q-	2 abnormalities	None
15	F/64	A	5.77	M	13q-	Normal	None
16	M/70	A	9.04	nd	13q-	Normal	None
17	M/75	A	13.36	M	13q-	Normal	None
18	F/67	A	20.08	nd	13q-	Normal	None
19	F/70	A	9	nd	13q-	Normal	None
20	F/79	A	10.12	nd	13q-	Normal	None
21	F/54	B	41.02	nd	Tri 12	1 abnormality	None
22	M/76	A	5.99	M	13q-	Normal	None
23	M/68	B	70.97	nd	11q-	Complex	None
24	F/84	A	20.44	nd	Normal	Normal	None
25	M/75	B	184.01	UM	Tri 12	1 abnormality	None
26	F/82	A	8.8	nd	13q-	Normal	None
27	F/85	A	71,98	nd	Normal	2 abnormalities	None
28	F/74	A	3.44	nd	13q-	1 abnormality	None
29	M/76	A	14.46	nd	13q-	Normal	None
30	F/85	A	19.51	M	13q-	Normal	None
31	F/52	A	11.9	nd	13q-	Normal	None
32	F/68	B	70.14	nd	11q-, 13q-	Complex	None
33	F/57	A	10.49	nd	13q-	Normal	None
34	F/80	B	35.01	nd	11q-	1 abnormality	None
35	M/62	A	11.42	nd	Normal	Normal	None
36	M/61	B	78	nd	13q-, Tri 12	Complex	None
37	F/53	A	15.58	UM	13q-	2 abnormalities	None
38	F/80	B	30.97	nd	11q-	1 abnormality	BR/relapse (month 12)
39	F/78	A	10.38	M	13q-	1 abnormality	RCb/relapse (month 48)
40	M/76	B	68.25	nd	17p-, 13q-	2 abnormalities	Ibrutinib/relapse (month 12)
41	M/80	B	11.06	UM	17p-, 13q-	Complex	Ibrutinib/relapse (month 24)
42	M/74	B	7.5	nd	nd	nd	FCR/relapse (month 96)
43	M/81	C	7.02	M	11q-, 13q-	nd	Alemtuzumab/relapse (month 72)
44	M/82	C	6.42	UM	11q-, 13q-	Complex	BR/relapse (month 24)
45	M/88	C	0.87	UM	17p-, 13q-	Normal	RCb/remission (month 8)
46	M/79	B	1.3	UM	Normal	Normal	BR/remission (month 8)
47	M/64	A	1.15	UM	Tri 12	1 abnormality	FCR/remission (month 54)
48	F/75	B	1.39	M	13q-, Tri 12	1 abnormality	FCR/remission (month 36)
49	F/64	B	0.26	UM	13q-	Normal	FCR/remission (month 10)
50	M/48	C	43.64	UM	17p-	Complex	Ibrutinib/remission (month 2)
51	F/72	B	20.2	UM	17p-, Tri 12	Complex	Ibrutinib/remission (month17)
52	M/66	C	7.64	UM	13q-, Tri 12	Complex	Ibrutinib/remission (month 2)

Patients were diagnosed according to the IWCLL criteria and staged according to the Binet system [19]. Newly diagnosed patients P28-P37. The patient cohort listed in this table is based in part on the cohort previously published by our group [18]. Fluorescent in situ hybridization (FISH) detecting 13q14 deletion (13q-), 11q22 deletion (11q-), 17p13 deletion (17p-) and trisomy 12 (Tri 12); chromosomal abnormalities are defined by conventional karyotyping [18,19]. *IGHV* mutational status data were not available for 26 patients, FISH not available for 2 patients, karyotype not available for 3 patients; not done (nd). Bendamustine and rituximab (BR). Rituximab and chlorambucil (RCb). Fludarabine, cyclophosphamide and rituximab (FCR). Alemtuzumab is a CD52 mAb. G/L, giga per liter; unmutated (UM); mutated (M).
